# The cell-type–specific genetic architecture of chronic pain in brain and dorsal root ganglia

**DOI:** 10.1172/JCI197583

**Published:** 2025-10-07

**Authors:** Sylvanus Toikumo, Marc Parisien, Michael J. Leone, Chaitanya Srinivasan, Huasheng Yu, Asta Arendt-Tranholm, Úrzula Franco-Enzástiga, Christoph Hofstetter, Michele Curatolo, Wenqin Luo, Andreas R. Pfenning, Rebecca P. Seal, Rachel L. Kember, Theodore J. Price, Luda Diatchenko, Stephen G. Waxman, Henry R. Kranzler

**Affiliations:** 1 Mental Illness Research, Education and Clinical Center, Crescenz VAMC, Philadelphia, Pennsylvania, USA.; 2 Center for Studies of Addiction, University of Pennsylvania Perelman School of Medicine, Philadelphia, Pennsylvania, USA.; 3 Alan Edwards Centre for Research on Pain,; 4 Department of Anesthesia, Faculty of Medicine and Health Sciences, and; 5 Faculty of Dental Medicine and Oral Health Sciences, McGill University, Montreal, Quebec, Canada.; 6 Department of Computational Biology, School of Computer Science, Carnegie Mellon University, Pittsburgh, Pennsylvania, USA.; 7 Medical Scientist Training Program, University of Pittsburgh School of Medicine, Pittsburgh, Pennsylvania, USA.; 8 Department of Neuroscience, Perelman School of Medicine, University of Pennsylvania, Philadelphia, Pennsylvania, USA.; 9 Center for Advanced Pain Studies, Department of Neuroscience, University of Texas at Dallas, Richardson, Texas, USA.; 10 Department of Neurological Surgery, and; 11 Department of Anesthesiology and Pain Medicine, University of Washington, Seattle, Washington, USA.; 12 Neuroscience Institute, Carnegie Mellon University, Pittsburgh, Pennsylvania, USA.; 13 Department of Neurobiology,; 14 Pittsburgh Center for Pain Research, and; 15 Department of Otolaryngology, University of Pittsburgh School of Medicine, Pittsburgh, Pennsylvania, USA.; 16 Veterans Affairs Connecticut Healthcare System, West Haven, Connecticut, USA.; 17 Department of Neurology, Yale University School of Medicine, New Haven, Connecticut, USA.

**Keywords:** Cell biology, Genetics, Neuroscience, Genetic variation, Molecular pathology, Pain

## Abstract

Chronic pain is a complex clinical problem comprising multiple conditions that may share a common genetic profile. GWAS have identified many risk loci whose cell-type context remains unclear. Here, we integrated GWAS data on chronic pain with single-cell RNA-Seq (scRNA-Seq) data from human brain and dorsal root ganglia (hDRG) and single-cell chromatin accessibility data from human brain and mouse dorsal horn. Pain-associated variants were enriched in glutamatergic neurons, mainly in the prefrontal cortex, hippocampal CA1-3, and amygdala. In hDRG, the hPEP.TRPV1/A1.2 neuronal subtype showed robust enrichment. Chromatin accessibility analyses revealed variant enrichment in excitatory and inhibitory neocortical neurons in the brain and in midventral neurons and oligodendrocyte precursor cells in the mouse dorsal horn. Gene-level heritability in the brain highlighted roles for kinase activity, GABAergic synapses, axon guidance, and neuron projection development. In hDRG, implicated genes were related to glutamatergic signaling and neuronal projection. In cervical DRG of patients with acute versus chronic pain, scRNA-Seq data from neuronal or non-neuronal cells were enriched for chronic pain–associated genes (e.g., *EFNB2*, *GABBR1*, *NCAM1*, *SCN11A*). This cell-type–specific genetic architecture of chronic pain across central and PNS circuits provides a foundation for targeted translational research.

## Abstract 2

Chronic pain genetics analyzed via GWAS and single-cell data revealed variant enrichment in brain glutamatergic and DRG C-fiber neurons, informing translational research pathways

## Graphical abstract

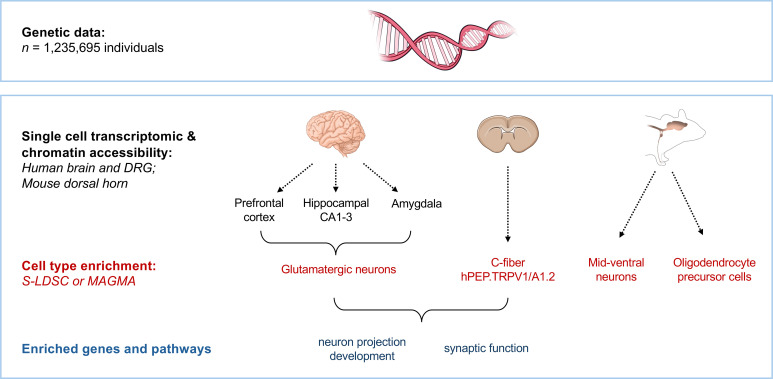

## Introduction

Chronic pain is both a symptom and a primary disease. It affects approximately 20% of the global population and is associated with psychiatric and physical comorbidities (1), contributing to high rates of morbidity and mortality (2). Despite its substantial and growing impact on public health (3), most currently available treatments for chronic pain are nonspecific, of limited efficacy, and associated with substantial adverse effects, including addiction (4–6). A deeper understanding of pain biology is crucial for the development of novel, targeted, safe, and effective treatments (7).

Integration of genomic data into drug development pipelines can yield drug approval rates that are 2.6 times those that lack such information (8) and could expedite the prioritization of new targets for chronic pain. Despite their diverse phenotypic manifestations, pain conditions exhibit high genetic overlap (9), a common genetic risk profile (10, 11), and similar associated alterations of the CNS (12, 13). GWAS have revealed hundreds of genetic risk loci for chronic pain, which account for a substantial fraction of the trait’s heritability (14–16). Previous GWAS of pain have shown that genetic loci located near genes expressed in the brain display enriched heritability (14, 17, 18), and more so for chronic pain than acute pain (19). However, despite the availability of transcriptomics data from various isolated brain regions, the cellular diversity of these regions remains unexplored.

Chronic pain can be caused and maintained by alterations in various components of the pain pathway, including dorsal root ganglia (DRG), the spinal cord dorsal horn (20, 21), and supraspinal brain centers (12, 13), with neuronal architecture organized into complex, hierarchically structured clusters of distinct cell types (22, 23). We recently showed that genetic variation that influences pain intensity is enriched in GABAergic neurons in the mid-brain (16) and identified a role for mouse dorsal horn neuron subtype–specific open chromatin in multisite chronic pain (24). While these findings relate to individual pain phenotypes with substantial genetic overlap (16), the shared downstream biological effects of the implicated genes on the complex cellular structure of the brain and dorsal horn are unidentified.

We recently performed a large GWAS meta-analysis of chronic pain phenotypes (*n* = 1,235,695) that implicated 343 genomic loci and revealed pleiotropic associations with psychiatric disorders, immune traits, and brain structures (25). Although this study revealed key genetic risk factors for chronic pain, the specific cell types that define the genetic associations underlying the cellular and circuit-based mechanisms (26, 27) — key to therapeutic targeting (28, 29) — are yet to be determined.

**Figure 1:**
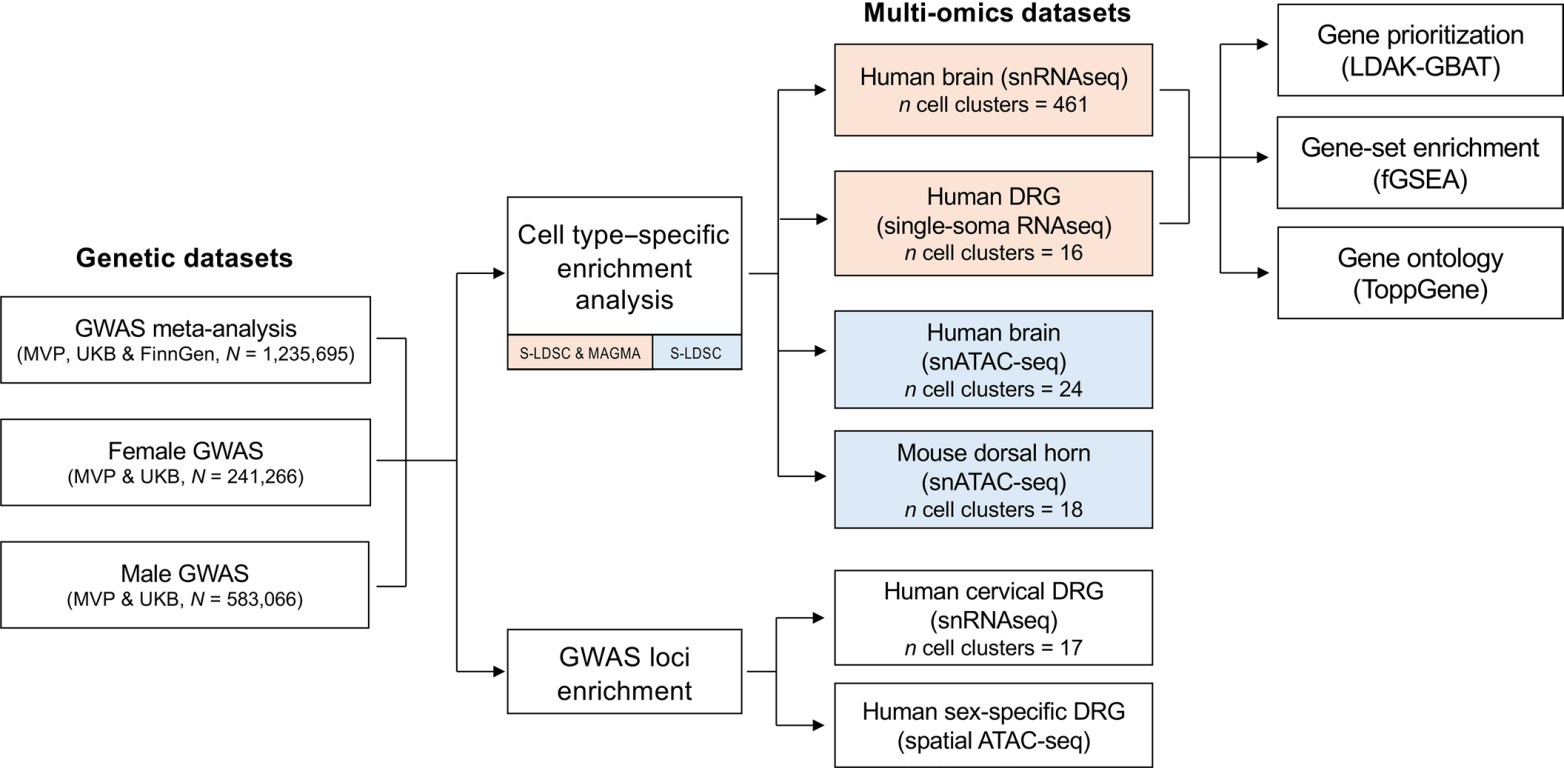
Study overview. Using S-LDSC, we first identified cell types enriched for chronic pain SNP heritability across single-nucleus transcriptomics data from human brain and hDRG, as well as chromatin accessibility data from human brain and mouse dorsal horn. To test the robustness of cell-type enrichment results in transcriptomic human brain and hDRG transcriptomics data, we used MAGMA, followed by gene prioritization and ontology analysis. Multi-omics datasets included in both S-LDSC and MAGMA analyses are shown in pink, while those included only in MAGMA are shown in blue. Finally, genetic loci from the pain meta-analysis were tested for enrichment in DEGs (snRNA-Seq) and sex-stratified GWAS for genes linked to differential chromatin accessibility (spatial ATAC-Seq) from hDRG datasets.

Here, we integrate multiple layers of single-cell omics from the brain, dorsal horn, and DRG with our GWAS meta-analysis of chronic pain to identify cell types and pathways relevant to the trait (Figure 1). This effort, made possible with datasets from 2 recent studies, advances our understanding of the physiological relevance of genomic associations for chronic pain. The first of these studies analyzed a comprehensive human brain transcriptomics dataset, using single-nucleus RNA-Seq (snRNA-Seq) of 3.369 million nuclei from 106 anatomical dissections across 10 brain regions (30). The second study was of the neural basis of human somatosensation that used single-soma RNA-Seq data from human DRG (hDRG) neurons (31).

## Results

### Cell-type identification using S-LDSC.

**Figure 2:**
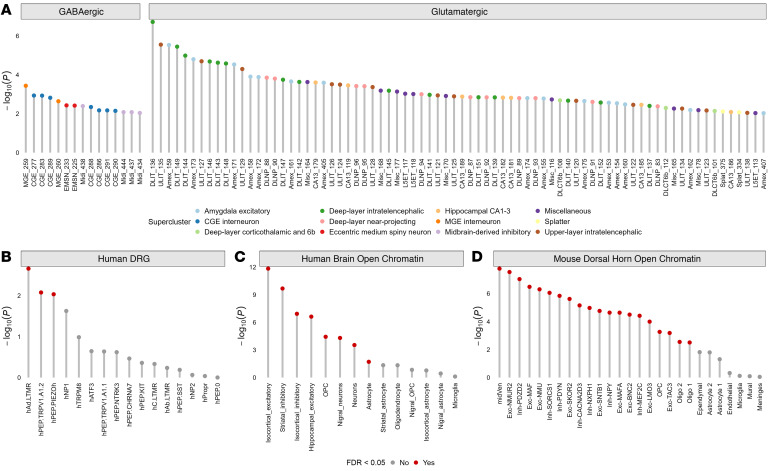
Cell-type enrichments for chronic pain. Cell-type numbers are denoted on the *x* axis, and statistical significance is shown on the *y* axis [–log_10_(*P*)]. (**A**) The significantly enriched cell types for chronic pain (*n* = 91, FDR *P* < 0.05) among 461 human brain cell clusters. Each cell type is colored according to its supercluster, and cell types are grouped into GABAergic (*n* = 15) or glutamatergic (*n* = 76) on the basis of their neurotransmitter annotation. See Supplemental Table 1 for full results. (**B**) Human single-soma DRG cell-type enrichment. (**C**) Human brain open chromatin cell-type enrichment. (**D**) Mouse dorsal horn open chromatin enrichment. Cell-type enrichments denoted by red in **B**–**D** are significantly enriched for chronic pain (FDR-corrected *P* < 0.05).

The brain’s cellular diversity has a hierarchical structure, with a foundational classification into broad categories (excitatory neurons, inhibitory neurons, neuromodulatory neurons and non-neuronal cells) that extends to more intricate superclusters of cells subdivided into clusters and subclusters or cell types (Figure 2) (22, 32). Applying stratified linkage disequilibrium score (S-LDSC) regression (33), we used this classification to assess chronic pain SNP heritability in 461 neuronal cell clusters generated from the extensive brain snRNA-Seq data (30). The 461 cell clusters were further organized into the broad neuronal types of GABAergic (inhibitory, *n* = 131) and glutamatergic (excitatory, *n* = 209) subtypes, of which 15 (~11.5%) and 76 (~36.4%), respectively, were significantly associated with chronic pain at the FDR 5% level (Supplemental Table 1; supplemental material available online with this article; https://doi.org/10.1172/JCI197583DS1).

Hypergeometric tests of neurotransmitter annotations for the associated cell types revealed significant enrichment of glutamatergic — but not GABAergic — neurons in chronic pain heritability within the brain (glutamatergic *P* < 9 × 10^–18^; GABAergic *P* = 0.9). The enriched glutamatergic neurons include 25 intratelencephalic clusters and 11 deep layers from the prefrontal cortex; 11 amygdalar excitatory neuronal cell types; 7 hippocampal neuronal types annotated to subfield CA1-3; three cortex-wide annotations to deep-layer 6b; and 2 “splatter” neuronal types, among others (Figure 2A and Supplemental Table 1). These enriched cell types were significantly overrepresented in the prefrontal cortex (*P* < 4 × 10^–24^), amygdala (*P* < 2 × 10^–4^), and hippocampus (*P* < 0.01).

The entire body is innervated by DRG neurons, which serve as the primary origin of the pain pathway (34). Thus, we explored peripheral neuronal cell types for chronic pain using single-soma hDRG data (31). Of the 16 hDRG cell types analyzed, 3 (hAδ.LTMR, hPEP.TRPV1/A1.2, and hPEP.PIEZOh) showed significant enrichment for chronic pain SNP heritability (FDR *P* < 0.05, Figure 2B and Supplemental Table 2).

Whereas studies support the enrichment of pain risk loci for mouse gene sets related to excitatory and inhibitory cellular pathways (35), we examined whether the SNP heritability of human chronic pain shows similar cell-type enrichment. To do so, we used S-LDSC (33) to partition the heritability of the chronic pain loci in putative *cis*-regulatory elements (CREs) from mouse dorsal horn neurons and glial cell types (24) and human brain neurons and glial cell types (36) (see Methods). We found significant enrichment for pain among brain neurons aggregated across subtypes of excitatory (hippocampal and isocortical) and inhibitory (striatal and isocortical) neurons (Figure 2C and Supplemental Table 3). Of the brain glial cell types, the putative CREs of astrocytes that are found across the neocortex, hippocampus, striatum, and substantia nigra were significantly enriched in chronic pain-associated genetic variation (Figure 2C). Consistent with prior evidence (24), we observed a broad enrichment of chronic pain variants across open chromatin of mouse spinal cord midventral neurons, inhibitory and excitatory neurons, and oligodendrocyte precursor cells (OPCs) (Figure 2D and Supplemental Table 4).

### Cell-type identification using MAGMA.

**Figure 3:**
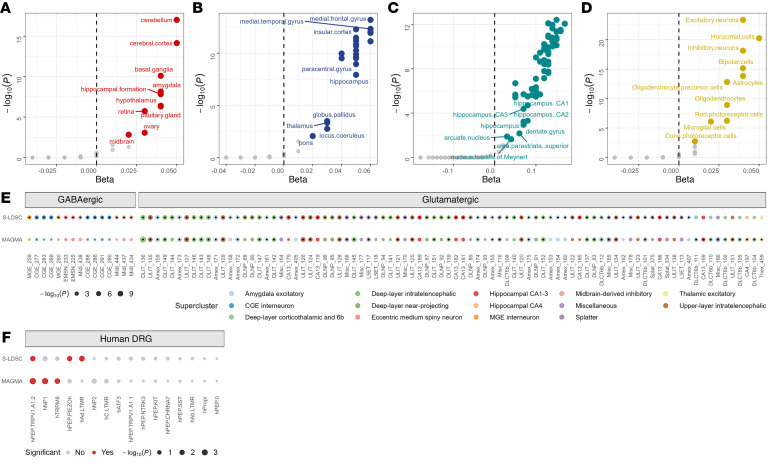
Comparison of MAGMA and S-LDSC cell-type enrichment analysis. (**A**–**D**) MAGMA-based gene enrichment results. Significant heritability enrichments (FDR *P* < 0.05) are uniquely colored for each tissue- and cell-level analysis. Tissue-based enrichment analysis used data from (**A**) CONSENSUS, (**B**) FANTOM, and (**C**) the Human Protein Atlas. (**D**) Cell-level enrichment analysis using RNA-Seq data from the Human Protein Atlas. (**E**) Combined MAGMA and S-LDSC results of significantly enriched cell types for chronic pain (101 unique cell types: 91 in S-LDSC versus 47 in MAGMA; FDR *P* < 0.05) among 461 brain cell clusters. Each cell type is colored according to its supercluster, and cell types are grouped into GABAergic (*n* = 15) and glutamatergic (*n* = 86) on the basis of their neurotransmitter annotation. Cell types that are significantly associated are marked by a black dot centered within each colored circle. In total, 37 cell types overlap across both methods. See Supplemental Tables 1 and 7 for full results. (**F**) Human single-soma DRG cell-type enrichment. Cell-type enrichments denoted by red in **F** are significantly enriched for chronic pain (FDR *P* < 0.05). In total, 1 cell type overlaps across both methods. See Supplemental Tables 2 and 8 for full results.

We used multi-marker analysis of genomic annotation (MAGMA) (37) to determine cell-type–specific enrichment of chronic pain gene-level associations. As expected, chronic pain variants mapped to the nearest gene were enriched largely in the brain (Figure 3A) and in neural subregions that process pain information (38, 39), such as the parietal, posterior insular, entorhinal, and medial frontal cortices, and the temporal gyrus (Figure 3, B and C, and Supplemental Table 5). We also observed robust enrichment in excitatory and inhibitory neurons, astrocytes, OPCs, and microglia (Figure 3D and Supplemental Table 6). Although immune cell types, including plasmacytoid DCs, naive B cells, and immune progenitor cells, were nominally enriched for chronic pain (*P* < 0.05), none survived multiple test correction (Supplemental Table 6).

Consistent with the S-LDSC analysis, we also investigated the enrichment for chronic pain in 461 cell-type clusters from the brain snRNA-Seq data (30) and 16 cell types from single-soma RNA-Seq hDRG data (31). Among the 461 brain cell-type clusters, only glutamatergic cell types (47 of 109; 22.5%) were significantly associated with chronic pain (FDR *P* < 0.05), all of which showed strong enrichment (hypergeometric *P* < 9 × 10^–18^) (Figure 3E and Supplemental Table 7). The cell types with the most enrichments (*n* = 29) were predominantly intratelencephalic neurons from the prefrontal cortex (*P* < 3.8 *×* 10^–2^). Other significant cell types were excitatory, including 7 cortex-wide annotations to deep-layer 6b (*P* < 3.5 *×* 10^–2^); eight amygdalar neuronal cell types (*P* < 3.3 *×* 10^–2^); two hippocampal neuronal cell types (annotated to subfields CA1-3, *P* = 3.2 *×* 10^–2^ and CA4, *P* = 2.2 *×* 10^–2^); and one thalamic excitatory neuronal cell type (*P* = 9.5 *×* 10^–3^) (Figure 3E and Supplemental Table 7). Of the 16 hDRG cell types analyzed, we observed enrichment for hPEP.TRPV1/A1.2, hNP1, and hTRPM8 (Figure 3F and Supplemental Table 8).

Following prior studies (40), we required that both our primary analytic method (S-LDSC) and the MAGMA gene property analysis provide strong evidence linking chronic pain genome-wide associations to specific cell types in the brain and hDRG. These methods rely on different assumptions and modeling approaches: S-LDSC is SNP based and evaluates GWAS heritability enrichment in the most cell-type–specific genes (33), whereas MAGMA uses proximity-based SNP-to-gene mapping and tests whether gene-level associations increase linearly with cell-type–specific expression (41). To reduce false-positives, we required both methods — each accounting differently for confounders such as gene size and linkage disequilibrium — to yield consistent results after multiple testing correction. Using this approach, we found that glutamatergic neurons — across 37 cell-type clusters — were enriched in chronic pain, primarily in cortical interneurons, the hippocampus, and the amygdala (Figure 3E). In the hDRG, both methods identified enrichment of the C-fiber thermoreceptor and nociceptor subtype hPEP.TRPV1/A1.2 (31) (Figure 3F).

### Contrasting cell-type enrichment for pain-related conditions.

**Figure 4:**
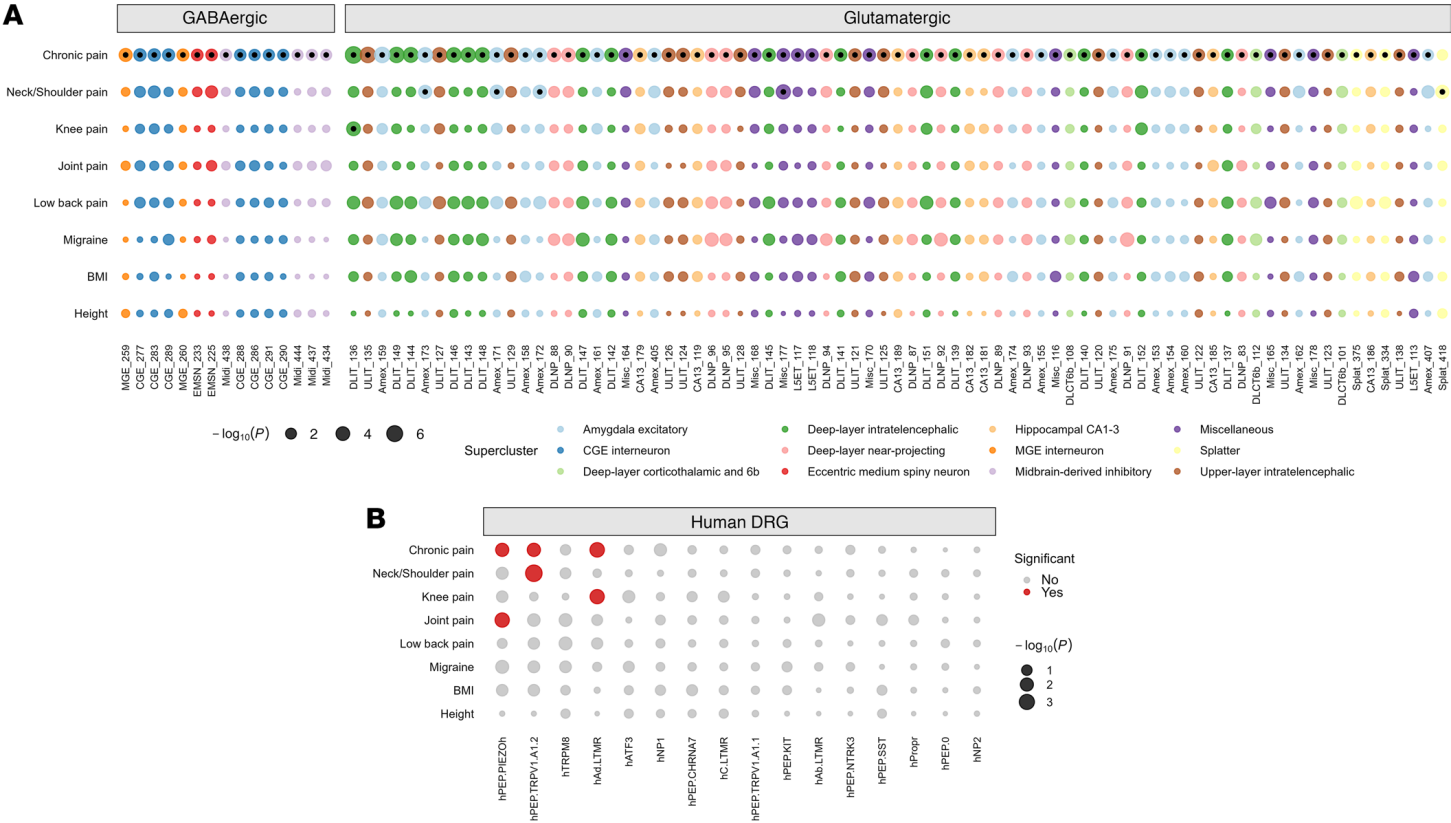
Cross-trait S-LDSC cell-type enrichment analysis. (**A**) Significantly enriched cell types across traits (chronic pain, *n* = 91; neck/shoulder pain, *n* = 5; knee pain, *n* = 1; FDR *P* < 0.05) among 461 brain cell clusters. Each cell type is colored according to its supercluster, and cell types are grouped into GABAergic (*n* = 15) and glutamatergic (*n* = 77) on the basis of their neurotransmitter annotation. Cell types that are significantly associated are marked by a black dot centered within each colored circle. In total, 4 significant brain cell clusters (DLIT_136, Amex_171, Amex_172, and Amex_172) overlap across traits. (**B**) Human single-soma DRG cell-type enrichment. Cell-type enrichments denoted by red in **B** are significantly enriched for chronic pain (FDR *P* < 0.05). All 3 significantly enriched hDRG cell types overlap in at least 2 traits. See Supplemental Tables 9 and 10 for full results.

To test for the presence of cellular heterogeneity across pain conditions, we used S-LDSC to link brain and hDRG data with GWAS of other pain-related phenotypes, including knee pain, neck/shoulder pain, joint pain, low back pain, and migraine (Figure 4).

The significantly enriched brain cell type for knee pain was an intratelencephalic glutamatergic neuron (DLIT_136, *P* = 4.3 *×* 10^–2^), consistent with previous findings of cortical glutamatergic neuronal activity in knee pain (42). For neck/shoulder pain, 5 cell types showed significant enrichment, largely in amygdalar excitatory neuronal cell types (e.g., Amex_171, *P* = 3.1 *×* 10^–2^, Amex_172, *P* = 3.1 *×* 10^–2^, and Amex_173, *P* = 3.1 *×* 10^–2^). This finding is consistent with evidence that links amygdalar hyperactivity with neck/shoulder pain (28, 43). Most (5 of 6) cell types were also significantly enriched in the chronic pain meta-GWAS results, highlighting a potential cross-trait pleiotropic cellular etiology (Figure 4A and Supplemental Table 9). No cell types for joint pain, low back pain, or migraine survived multiple testing corrections (Supplemental Table 9).

In the hDRG, hPEP.TRPV1/A1.2 was significantly enriched for neck/shoulder pain (*P* = 2.5 *×* 10^–3^), consistent with the role of C-fiber thermoreceptors and nociceptors in pain sensation (31). Joint pain was enriched for hPEP.PIEZOh (*P* = 4.3 *×* 10^–2^), suggesting a role for neurons that express high levels of *PIEZO2* (31, 44). The significantly enriched hDRG cell type for knee pain was hAδ.LTMR (*P* = 4.6 *×* 10^–2^), highlighting its potential role in tactile sensation and pain modulation in the context of knee pain (45). Notably, all 3 cell types that showed significant enrichment across these traits were also enriched in S-LDSC results for the chronic pain meta-GWAS (Figure 4B). At a FDR of *P* of less than 0.05, no hDRG cell types were significantly enriched for low back pain or migraine (Figure 4B and Supplemental Table 10), which is expected for migraine, since this is a disorder of the trigeminal ganglia (TG) system, and there are important differences between the DRG and the TG (46, 47).

### Cell-type enrichment for chronic pain in males and females.

Sex differences could play a role in pain neurobiology (48, 49). There is emerging evidence of sex-specific enrichment of pain GWAS loci/genes in the human brain (18) and DRG (49, 50) and in mouse DRG (51). However, the cell-type–specific context needed to clarify these enrichment patterns in the human brain and hDRG are yet to be determined. We thus partitioned the heritability of sex-stratified pain GWAS loci (25) in the brain and hDRG. In male individuals, we identified significant SNP heritability signals for chronic pain in 10 GABAergic and 65 glutamatergic (VGLUT1, VGLUT2, and VGLUT3) brain neuronal cell types. Looking at the broad neuronal types, glutamatergic — but not GABAergic — neurons were significantly enriched for chronic pain (hypergeometric *P* < 6 × 10^–17^). Among females, only the intratelencephalic glutamatergic cell cluster (DLIT_136, *P* = 1.1 *×* 10^–2^) was significantly associated with chronic pain SNP heritability (Supplemental Figure 1A and Supplemental Table 11).

In the hDRG, hPEP.PIEZOh, hNP1, and hTRPM8 were nominally significant (*P* < 0.05) cell types among females, and hPEP.TRPV1/A1.2, hAδ.LTMR, and hTRPM8 were nominally significant among males, though none of these cell types was significantly enriched after correction for multiple testing (Supplemental Figure 1B and Supplemental Table 12).

### Prioritizing heritable genes for chronic pain in the brain and DRG cell types.

We extended our investigation using LDAK-GBAT (52), fast gene set enrichment analysis (fGSEA) (53), and ToppGene (54) analyses to understand the biological mechanisms of chronic pain cell-type enrichments. These analyses enabled us to estimate the chronic pain heritability attributable to individual genes (Supplemental Table 13), pinpoint heritable gene clusters in the brain and hDRG, and identify biological processes and pathways enriched for the gene clusters in each cell type.

**Figure 5:**
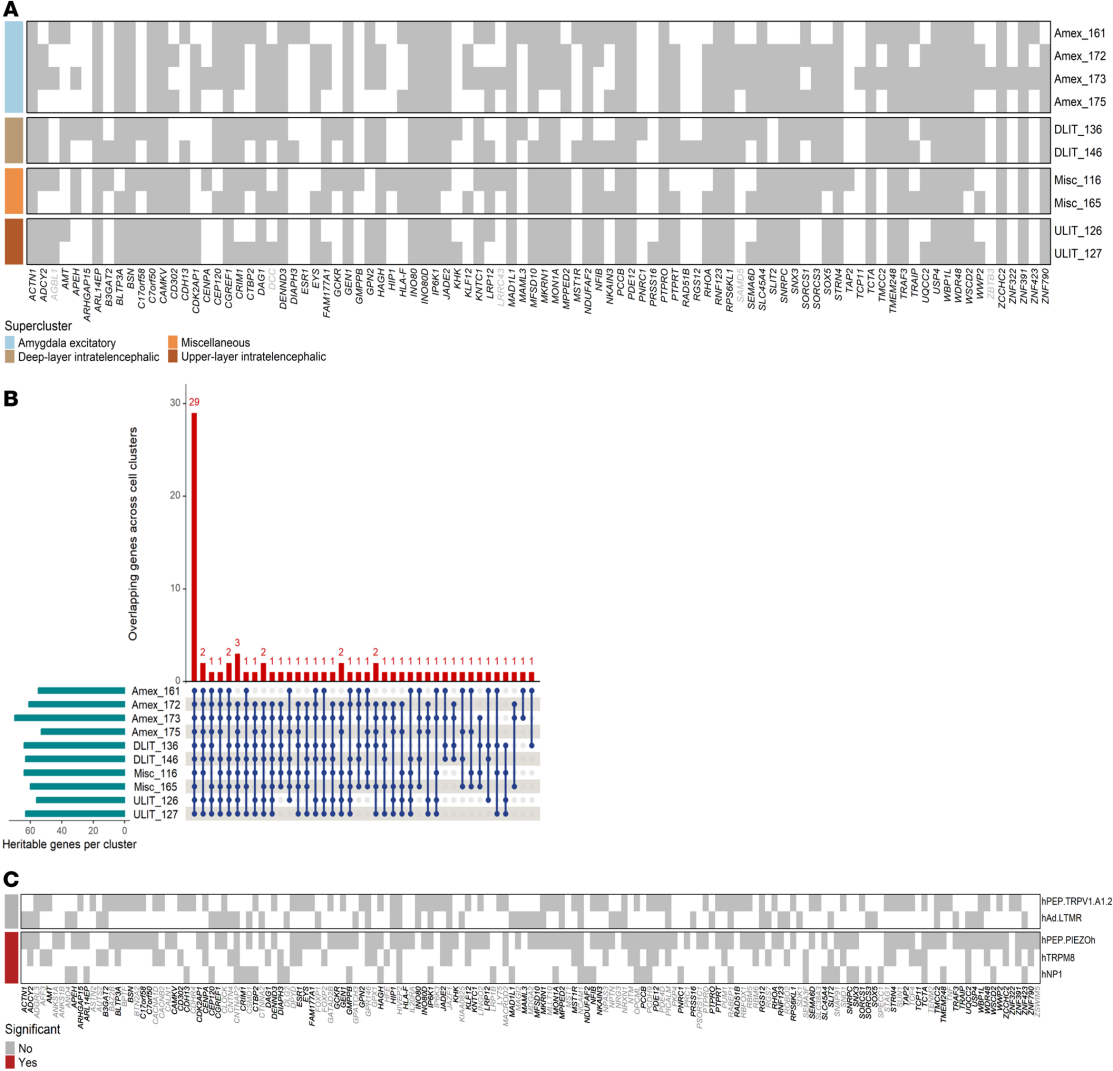
Heritable genes in brain and hDRG cell types. (**A**) The significantly heritable genes among 10 enriched brain cell clusters. Each cell type is colored according to its supercluster. (**B**) Gene overlap across brain cell types (**C**) Significantly heritable genes among 5 associated hDRG cell clusters. Overlapping genes in brain (**A**) and hDRG (**C**) are shown in black.

Among the 37 glutamatergic neuronal types significantly enriched for chronic pain in the brain (Figure 3E), heritable gene clusters were enriched in 25 neuronal clusters (nominal *P* < 0.05), ten of which were significant after multiple testing correction (FDR *P* < 0.05) (Figure 5A and Supplemental Table 14). Of the 94 heritable genes identified in the 10 cell types, 29 were shared among them (e.g., *ACTN1*, *BSN*, *DAG1*, *SLC45A4*, and *PTPRO*) (Figure 5B). In the hDRG, hPEP.PIEZOh, hNP1, and hTRPM8 were nominally significant (*P* < 0.05), although none survived multiple testing (FDR *P* > 0.05) (Supplemental Table 15). Notably, 89 of 94 (~94.7%) of the enriched genes in the brain cell types were also identified in hDRGs (Figure 5, A and C).

Gene overrepresentation analysis using heritable genes from the 10 significant brain cell types revealed enrichment for neuron projection, axon guidance, and synapse-related processes (Supplemental Figure 2 and Supplemental Table 16). Particularly noteworthy is the intratelencephalic cell type ULIT_127, with heritable genes enriched for protein kinase activity (*PTPRO* and *PTPRT*), GABAergic synapse (*BSN*, *CDH13*, *CTBP2*, *DAG1*, and *PTPRO*), dorsal/ventral horn axon guidance (*DCC* and *SLIT2*), and neuron projection (*DCC*, *PTPRO*, *RHOA*, *SLIT2*, and *SEMA6D*) (Supplemental Table 16). In hDRG, prioritized heritable genes for hPEP.TRPV1/A1.2 were implicated in kinase activity (*IP6K3* and *IP6K1*), calcium channel activity (*CACNA1D* and *CACNB2*), glutamatergic synapse (e.g., *BSN*, *MDGA2*, *NPTN*, *NRXN1*, *PTPRD*), somatodendritic compartments (e.g., *ACTN1, BSN, CACNA1D, CNTNAP2, MAGI2, NCAM1*), and neuron projection (e.g., *AUTS2*, *CTNNA2*, *CNTNAP2*, *NCAM1*, *NRXN1*, *PTPRO*), among others (Supplemental Figure 2 and Supplemental Table 17).

In summary, gene clusters with heritable links to chronic pain in the brain and hDRG cell types converge on biological processes related to synaptic function and neuron projection.

### Identification of differentially expressed or accessible genes linked to chronic pain risk.

As in our current analyses, prior human pain GWAS studies that used S-LDSC and MAGMA analyzed transcriptomics data from healthy individuals or individuals with an unknown disease status (14, 15). However, in rodents, hundreds of genes are differentially expressed in the DRG in response to painful stimulus (55). At the same time, varying transcriptomics effects have been identified in the blood, spinal cord, and brain of humans experiencing pain (47, 56). Dysregulated genes are crucial for host immune defense and wound healing, as well as pain modulation, which enables the host to adopt survival behaviors (56, 57). We hypothesized that decoding the chronic pain GWAS at loci of pain-perturbed genes would explain a larger proportion of heritability than loci of homeostatic genes. To test this hypothesis, we selected differentially expressed genes (DEGs) (FDR *P* < 0.01) from a scRNA-Seq dataset of cervical DRG samples from patients with acute pain versus those with chronic pain (58). The heritability enrichment at these genetic loci was assessed via QQ plots, where the gene-level MAGMA-derived chronic pain GWAS *P* values were gauged against those expected by chance.

**Figure 6:**
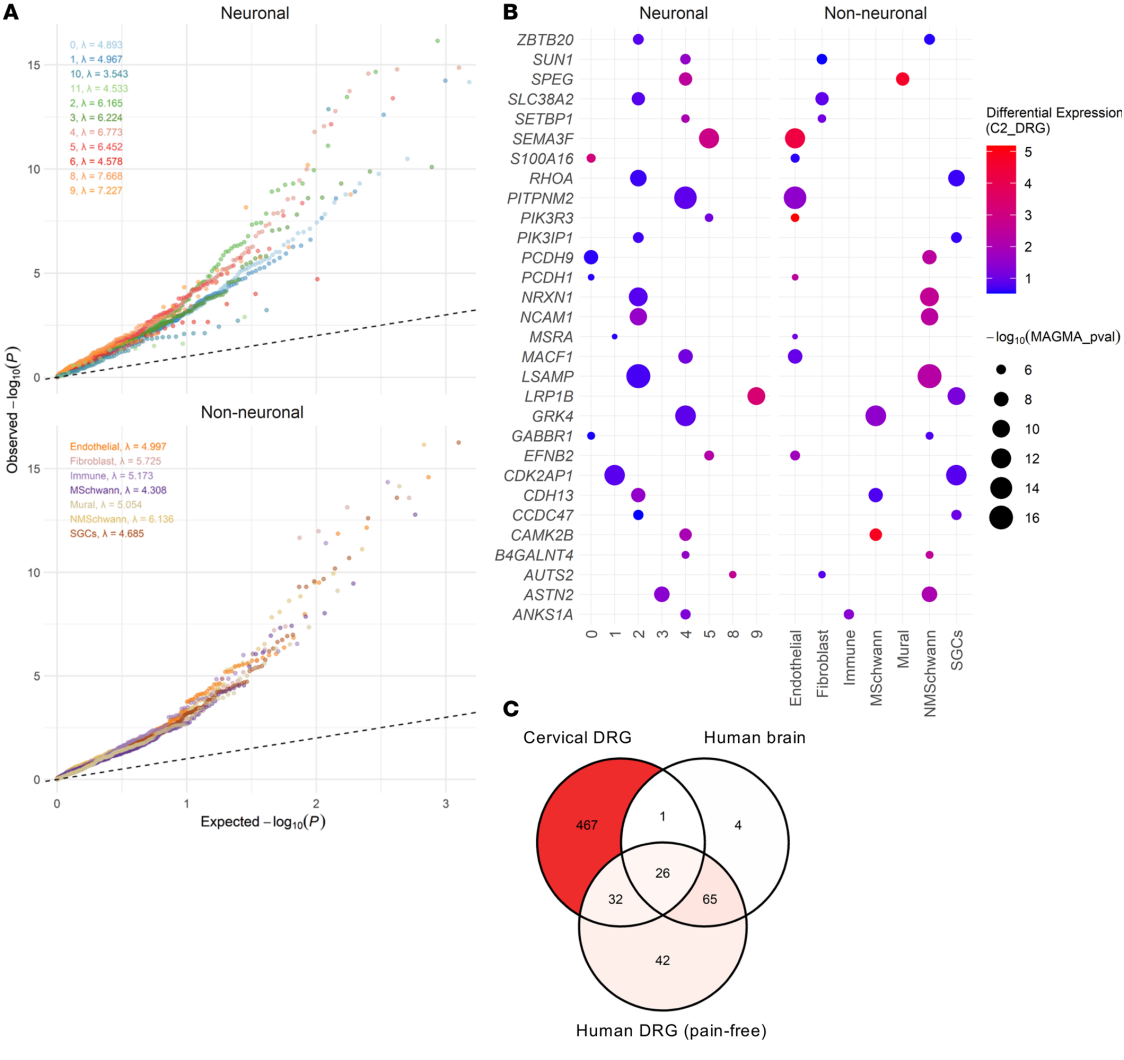
Chronic pain–associated genes in cervical DRG of patients with acute versus chronic pain. (**A**) QQ plots of pain-associated genes in neuronal and non-neuronal cervical DRGs. (**B**) The top 30 differentially expressed genes in neuronal and non-neuronal cervical DRG cell types that are associated with chronic pain risk. SGCs, satellite glial cells; NMSchwann, nonmyelinated Schwann; MSchwann, myelinated Schwann. (**C**) Gene overlaps between cervical DRGs and gene-level results from brain and hDRG (pain-free) cell types.

We found that DEGs in both neuronal and non-neuronal cell types had steep slopes (λ) in these QQ plots (neuronal cells: λ = 3.5 – 7.7; non-neuronal cells: λ = 4.3 – 6.1), indicating greater enrichment of significant *P* values for chronic pain than would be expected by chance (Figure 6A). Gene set enrichment between the sets of genes differentially expressed in individuals with acute or chronic pain and the MAGMA pain-associated genes provide substantial support for 526 genes, of which 88 were differentially expressed in at least 2 cell types (Supplemental Table 14). The top DEGs and those most strongly associated with findings from our chronic pain GWAS were *EFNB2* (*P*_MAGMA_ = 8.6 *×* 10^–7^), *GABBR1* (*P*_MAGMA_ = 6.2 *×* 10^–13^), *GRK4* (*P*_MAGMA_ = 6.2 *×* 10^–13^), *LSAMP* (*P*_MAGMA_ = 3.4 *×* 10^–34^), *NCAM1* (*P*_MAGMA_ = 2.5 *×* 10^–15^), *NRXN1* (*P*_MAGMA_ = 6.2 *×* 10^–13^), *PITPNM2* (*P*_MAGMA_ = 2.5 *×* 10^–15^), and *SEMA3F* (*P*_MAGMA_ = 2.5 *×* 10^–15^) (Figure 6B). We also found evidence of *SCN11A* loci enrichment in a neuronal cell cluster (*P*_MAGMA_ = 6.8 *×* 10^–4^, Supplemental Table 18). We observed an overlap between DEGs in cervical DRGs and heritable genes from the brain (27 of 95) (30) and pain-free hDRGs (58 of 164) (31) (Figure 6C).

Using the same approach as above, we also tested for enrichment of GWAS loci in differentially accessible regions (DARs) from a spatial assay for transposase-accessible chromatin using sequencing (ATAC-Seq) hDRG dataset comparing neuronal chromatin accessibility between sexes (59) (females: λ = 2.5; males: λ = 3.4; FDR *P* <  0.01; Supplemental Figure 3A). In females, hDRG DAR gene associations included genes encoding the netrin 1 receptor *DCC* and sodium transporter *SLC4A10* (Supplemental Figure 3B and Supplemental Table 19). In males, the top hDRG DAR genes most strongly enriched for chronic pain genetic signals were involved in kinase activity (*CAMKV*, *DCAKD*), calcium signaling (*ERBB3*, *ITPR3*), and synaptic function (*BSN*) (Supplemental Figure 3B and Supplemental Table 19).

## Discussion

In this study, we investigated the cell-type context of genetic associations for chronic pain in the CNS and PNS. By integrating GWAS and single-cell omics data, we identified enrichments of glutamatergic neurons in the brain and peripheral enrichments for hDRGs in C-fiber neuronal subtype hPEP.TRPV1/A1.2. Analysis of chromatin accessibility revealed enrichment for pain in neocortical inhibitory and excitatory neurons in the human brain, and midventral neurons, inhibitory and excitatory neurons, and OPCs in the mouse dorsal horn.

Analysis of brain snRNA-Seq data using a broader chronic pain GWAS meta-analysis — which included our earlier pain intensity GWAS — revealed predominant enrichment in glutamatergic neurons, augmenting prior findings of GABAergic enrichment (16). Greater support for the role of glutamate here may reflect the greater resolution of our current dataset, which spans 461 cell types, substantially more than the 25 cell types previously assessed, potentially revealing unique biological patterns. Dysfunction in the metabolism of GABA and glutamate, resulting in varying profiles of their metabolites in brain, has been observed across different pain conditions (60, 61). Studies in mice have also shown suppression of glutamatergic activity in the ventral tegmental area (VTA), with enhanced VTA glutamatergic inputs to the prelimbic cortex, which diminish pain-like behaviors (62). Lower medial prefrontal cortical glutamate levels have also been seen in people with chronic pain (63). However, the genomic contribution of chronic pain variants to glutamatergic signaling in humans has been largely unsubstantiated. Thus, our finding that genes involved in chronic pain are enriched in intratelencephalic, hippocampal, and amygdalar excitatory neurons that express glutamatergic transporters (VGLUT1, VGLUT2, and VGLUT3) provides genomic insights into the molecular identity of key neuronal subtypes in the brain, consistent with aspects of the mesolimbic theory of pain (64).

Brain imaging studies show amygdalar activation in both acute and chronic pain, with meta-analyses localizing effects to the laterobasal amygdala, consistent with its role in the emotional dimensions of pain (61, 65). Patients with chronic pain also tend to exaggerate recalled pain intensity — a bias linked to posterior hippocampal morphology and reduced hippocampal neurogenesis (65). The medial prefrontal cortex (mPFC) integrates pain with mood and cognition, and altered glutamatergic signaling within cortico-limbic circuits (amygdala, hippocampus) contributes to maladaptive plasticity (66). Magnetic resonance spectroscopy studies reinforce these mechanisms: fibromyalgia and chronic low back pain are associated with elevated glutamate levels and reduced GABA in the anterior cingulate, insula, and mPFC (67–69). Together, these findings suggest that excitatory activity and disrupted cortico-limbic connectivity contribute to persistent pain. Our observations of gene enrichment across CNS regions — including the cortex, hippocampus, and amygdala — may therefore have important implications for pain therapeutics, highlighting the potential need for analgesic drugs that cross the blood-brain barrier into the CNS, a consideration that may be overlooked in early-stage drug development (70).

In the periphery, glutamatergic pain-signaling neurons detect noxious stimuli and transmit fast, sharp pain signals via Aδ-fibers and slow, dull pain via C-fibers (29). These first-order pseudounipolar neurons, with cell bodies in the DRG, synapse via glutamate on second-order neurons. In addition, DRG sensory neurons release neuropeptides that can act pharmacologically on spinal neurons, modulating the activity of second-order neurons and thereby influencing signals relayed to third-order neurons in the brain regions that process pain (71, 72). Musculoskeletal pain heritability has been mapped to both C-fiber peptidergic (PEP1) and nonpeptidergic (NP2) neuronal types in the macaque DRG (73). Extending these findings to humans, we show that chronic pain genetic associations are enriched in the C-fiber peptidergic subtype hPEP.TRPV1/A1.2 in hDRGs. Different DRG neuronal types may be enriched for pain in different bodily sites (74, 75). In macaques, PEP1 neurons are enriched for facial, neck/shoulder, stomach, and hip pain, and NP2 neurons for back and hip pain (73). Here, we observed that genetic signals contributing to joint, knee, and neck/shoulder pain are enriched in hPEP.PIEZOh, hAδ.LTMR, and hPEP.TRPV1/A1.2, respectively. Taken together, our results differentiate the DRG neurons that mediate genetic risk for pain in humans, implicating A-fiber mechanosensory channels, C-fiber thermo-nociceptors, and tactile sensation neurons (31).

Gene-level heritability and overrepresentation analysis in brain and hDRGs converge to show that neuronal development and differentiation and regulation of synaptic organization are key mechanisms of chronic pain. In cervical DRGs from patients with acute pain versus those with chronic pain (58), several chronic pain–associated genes (e.g., *EFNB2*, *GABBR1*, *GRK4*, *NCAM1*, and *NRXN1*) were differentially expressed across both neuronal and non-neuronal cell types. In contrast, differential expression of *SCN11A* was specific to neurons. These findings highlight the key molecular features of human sensory neurons across the PNS. Non-neuronal cells, including satellite glial cells and immune cells, become reactive after injury, releasing proinflammatory mediators and enhancing neuron/glia signaling (58, 76). Such interactions can modulate neuronal excitability and contribute to chronic pain (76), suggesting that GWAS signal enrichment in glial and immune genes in the cervical DRGs may underlie the role of these cells in genetic susceptibility to chronic pain.

As gene expression profiles are largely determined by distinct epigenomic signatures at gene promoters and enhancers, we explored enrichment for pain using single-nucleus ATAC-Seq (snATAC-Seq) data from the human brain and mouse dorsal horn. We identified putative *cis*-regulatory elements in brain excitatory (hippocampal, isocortical) and inhibitory (striatal, isocortical) neurons and astrocytes. In line with our recent work (24), we also found that chromatin accessibility in the spinal cord, involving superficial dorsal and midventral neurons, oligodendrocytes, and OPCs of the mouse dorsal horn was enriched for chronic pain. These findings shed light on the neural gene regulatory circuits and provide a framework for interpreting noncoding genomic variants linked to chronic pain. Cross-species comparisons strengthen this framework by providing access to chromatin accessibility data from spinal cord cell types, in which high-quality human data remain limited. The mouse analyses are particularly informative because many enhancers show evolutionary conservation (77), although species differences in circuitry and glial function (78) and the gap between reflexive rodent assays and the subjective human pain experience (79, 80) warrant caution. Nonetheless, the chromatin accessibility enrichment observed in both human and mouse datasets highlights conserved biology, while underscoring the need for validation in human tissue.

Our findings build upon previous studies that reported sex-specific tissue enrichment of pain-associated signals in the human brain (18) and DRG (49, 50) and mouse DRGs (51). Specifically, we characterized cell-type enrichment patterns for chronic pain in males and females separately and showed that glutamatergic (VGLUT1, VGLUT2, and VGLUT3) neuronal cell types were enriched among males, while only an intratelencephalic glutamatergic neuron was enriched in females. Extending our analysis to chromatin accessibility in hDRG neurons among the sexes, we found that female DAR-gene associations involved *DCC* and *SLC4A10*, whereas male-enriched genes were linked to kinase activity (*CAMKV*, *DCAKD*), calcium signaling (*ERBB3*, *ITPR3*), and synaptic function (*BSN*).

Our findings would be enhanced by the availability of additional information at several levels. First, although the genetic correlations among pain phenotypes in the meta-analysis were highly significant and positive, they were far from unity (MVP vs. UKB: *r*_g_ > 0.79, *P* < 2.23 × 10^−308^, MVP vs. FinnGen: *r*_g_ = 0.66, *P* = 1.30 × 10^−163^, and UKB vs. FinnGen: *r*_g_ = 0.69, *P* < 2.97 × 10^−213^). This likely reflects heterogeneity in the pain assessment methods across biobanks (i.e., quantitative in MVP and UKB vs. binary in FinnGen). MVP and UKB captured pain as ordinal measures that reflected chronicity, whereas FinnGen used a binary indicator of general pain without a defined time frame. Chronic pain is inherently variable because of differences in pain tolerance, assessment methods, and treatment effects (81, 82). Future GWAS should prioritize deep phenotyping to identify shared mechanisms and improve consistency across pain assessments in large, multi-biobank cohorts.

Second, the brain snRNA-Seq dataset (30), which comprises 461 cell-type clusters from 10 brain regions, although the most comprehensive to date, is incomplete. Other brain regions involved in nociceptive signaling, such as the VTA (62) and midbrain periaqueductal gray (83), are not represented in the snRNA-Seq data. Also, by using mouse orthologs of human open chromatin data, we lack information about human enhancers not found in mice. Third, snRNA-Seq and related omics datasets do not include individuals of non-European ancestry, limiting our ability to generalize the findings to a more diverse population. Furthermore, the greater brain cell-type enrichment for pain observed in males than females may be attributable to the predominance of males in the GWAS, primarily driven by the MVP cohort. Given the limited power, we did not perform formal SNP × sex interaction tests and therefore interpret these differences as descriptive rather than evidence of sex-specific mechanisms. We clearly indicate nominal cervical hDRG enrichments and acknowledge that statistical power and multiple testing correction constrain the strength of these findings. Larger, sex-balanced, and ancestrally diverse GWAS and transcriptomics data will be needed to rigorously assess sex differences.

Gene-based analysis revealed differential expression of *SCN11A* in cervical DRG neurons, but not of *SCN9A* or *SCN10A*. Given the established genetic links between *SCN9A* and *SCN10A* and pain (84–86), along with mechanistic evidence implicating Nav1.7-SCN9A, Nav1.8-SCN10A, and Nav1.9-SCN11A as key mediators of DRG neuron–driven pain signaling (87), the absence of association signals for *SCN9A* and *SCN10A* was unexpected. This discrepancy may reflect the strong contribution of Nav1.9-SCN11A to the control of excitability of both normal DRG neurons (88–90) and in disease states caused by channel mutations (86) or inflammation (91). Alternatively, it may reflect phenotypic dilution in the GWAS meta-analysis, which included relatively few true cases of neuropathic pain. A more targeted GWAS using a physiologically relevant phenotype — such as painful peripheral neuropathy — may capture more fully the genetic contributions to neuropathic pain and hDRG function. Another critical advancement would be to conduct the same analyses using developmental datasets (92, 93) to gauge the contribution of chronic pain genetic variation in human brain and DRG development.

Transcriptomics data from disease-free individuals have been useful for identifying disease-relevant tissues and cell types (94), but bulk tissue sequencing can miss signals specific to individual cell types. Single-cell sequencing overcomes this limitation, has been successfully applied to psychiatric disorders (95, 96), and was extensively used in our study. However, data from healthy individuals alone provide an incomplete view, as only comparisons between diseased and healthy states can reveal genes that are differentially regulated in pain-related pathways. The greater number of heritable genes identified in chronic versus acute pain in the cervical DRG snRNA-Seq data supports this point (Figure 6C). The overlap between gene-level chronic pain GWAS signals and genes differentially expressed in pain-perturbed human DRGs suggests functional relevance but does not necessarily imply causality. The thousands of DEGs typically observed in transcriptomics studies often reflect downstream effects of a small number of differentially expressed transcription factors, which are themselves regulated through complex molecular cascades (49). Thus, it is unclear which of these DEGs are causally involved in the molecular etiology of chronic pain. Definitive assessment would require gene-by-gene experimental validation, which is beyond the scope of this work. The field is ripe for large-scale, single-cell data collection across the pain matrix (nerves, DRG, spinal cord, brain) to identify pain-perturbed genes and enable high-resolution decoding of chronic pain GWAS findings.

In conclusion, our findings extend prior work by mapping genomic loci associated with chronic pain onto human brain and sensory neuronal types to identify its cellular origin. Genetic influences on pain identified in this study are predominantly enriched in glutamatergic neurons within the brain and in C-fibers of the hDRG. Heritable genes for pain in both cell types converge on synaptic function and neuronal development. Combined with gene enrichment analysis in cervical DRG that revealed enrichment of DEGs in neuronal and non-neuronal cell types, this study defines a broad set of central and peripheral neuronal features for genes associated with pain, offering a basis for translational studies.

## Methods

### Sex as a biological variable.

For sex-stratified analyses, we included summary statistics from our GWAS meta-analysis of chronic pain that combined sex-stratified GWAS of MVP pain intensity (male individuals, *n* = 404,510; female individuals, *n* = 32,173) (16) and UKB MCP (male individuals, *n* = 178,556; female individuals, *n* = 209,093) (51), to yield a total of 583,066 male individuals and 241,266 female individuals of European ancestry (25).

We obtained snATAC-Seq data from a study of the mouse dorsal horn that harvested single nuclei from lumbar segments L3–L5 from 10 mice (*n* = 5 females/*n* = 5 males, 7–8 weeks old), pooled them, and identified 74,437 glial cells and 19,073 neurons (24). Neurons were grouped into 18 species-conserved subtypes (24).

### GWAS data selection.

We included summary statistics from the largest GWAS meta-analysis to date of chronic pain (*n* = 1,235,695 individuals of European-like ancestry [EUR]) (25). The meta-analysis combined GWAS from 3 biobanks. The first GWAS was performed by us in the Million Veterans Program (MVP) (*n* = 436,683 EUR individuals) (16). This study examined pain intensity (as a proxy for chronic pain), represented by the median of the annual median pain ratings measured across multiple years with an 11-point ordinal numeric rating scale, a consistent and valid measure of self-reported pain (97, 98). The second GWAS was conducted in the UK Biobank cohort (UKB) (*n* = 387,649 EUR individuals) (14). That study examined multisite chronic pain (MCP), defined as the count of chronic pain reported across 7 bodily sites (i.e., head, face, neck/shoulder, back, stomach/abdomen, hip, knee), which yielded an 8-point ordinal score. The third GWAS was of “pain (limb, back, neck, head abdominally)” ascertained using International Classification of Diseases 9 (ICD-9) and ICD-10 codes (Finnish version) from the FinnGen cohort (data freeze 10; *n* = 411,363 EUR individuals) (99). The FinnGen study assessed overall pain, with cases (*n* = 189,683) required to have 1 or more ICD-9 or ICD-10 diagnostic codes for 16 disorders with a pain component (occurring in joints, limbs, neck, head, abdomen, and back) and none for controls (*n* = 221,680).

In addition to the chronic pain meta-GWAS, we selected 7 comparison phenotypes that were both polygenic and had been studied in large GWAS. These phenotypes were selected to address concerns about specificity and phenotypic and genetic pleiotropy across pain phenotypes. We used published GWAS data for knee pain (UKB only, *n* = 254,380) (10), neck/shoulder pain (UKB only, *n* = 237,825) (10), joint pain (UKB and FinnGen, *n* = 722,279), low back pain (UKB and FinnGen, *n* = 693,056), and migraine (UKB and FinnGen, *n* = 802,589) (99). Whereas questionnaires that examined localized pain lasting longer than 3 months were used in the UKB dataset (51), the FinnGen sample ascertained pain in the same bodily location as in UKB using ICD-9 or ICD-10 codes with no duration specified (99). We also included 2 phenotypes — height (*n* = 4,080,687) (100) and BMI (*n* = 339,224) (101) — as negative controls, as their genetic basis differs substantially from that of pain-related disorders.

### Multi-omics data selection.

To establish a broad biological enrichment pattern of the genetic associations for chronic pain, we downloaded bulk and scRNA-Seq data from 6 human tissues and cell types from the Human Protein Atlas (version 24) (102). These datasets comprise (a) 50 human tissues from GTEx (version 8) (103) and the Human Protein Atlas (version 24) (102); (b) 46 tissues from the FANTOM consortium (104); (c) 193 brain subregions from the Human Brain Tissue Bank of Semmelweis University (102, 105); (d) scRNA-Seq data from 81 cell types in the Human Protein Atlas (version 24) (102); (e) 19 immune cell types from the Human Protein Atlas (version 24) (102); and (f) 30 immune cell types from Monaco et al. (106). For data processing, gene expression levels measured in transcripts per million (TPM) were log transformed [as is typical for such data, we used log_2_(1 + TPM)] to compress the scale and reduce outliers, with the mean-transformed expression for each gene in each cell type retained for further analysis.

To measure cell-type enrichment of chronic pain in the brain and dorsal horn with high sensitivity, we obtained (a) snRNA-Seq data from a study of the transcriptomic diversity of cell types in approximately 100 locations across the human brain, which identified 461 cell-type clusters and 3,313 subclusters (30), and (b) single-soma RNA-Seq data from a study of human somatosensory physiology using hDRG, which identified 16 cell-type clusters (31). Both datasets are the most comprehensive human brain and hDRG snRNA-Seq data available to date, to our knowledge.

To examine chromatin accessibility enrichment patterns for chronic pain, we obtained snATAC-Seq data from a study of the human brain: 10 samples spanning the isocortex (*n* = 3), the striatum (*n* = 3), the hippocampus (*n* = 2), and the substantia nigra (*n* = 2), and 70,631 total cells (36).

### Calculation of cell-type expression specificity.

We created a gene expression specificity matrix for each cell type by regressing expression levels of gene features with cell-type specificity using a binary measure (examining whether the cell type is present [yes = 1] or absent [no = 0]) among the defined cell clusters, adjusted for the age and sex of the sample donors. For each cluster, genes were ordered by decreasing association test statistics (using gene expression as a marker of the cell type), with the top 1,000 retained for S-LDSC analyses (33).

### Heritability enrichment of chronic pain risk loci.

Using S-LDSC (33), we partitioned the SNP heritability for chronic pain and examined the enrichment of the partitioned heritability in both snRNA-Seq data from the adult human brain (30) and single-soma RNA-Seq data from the hDRG (31). Following a previously described LDSC pipeline (107), we also estimated conditional heritability enrichment of chronic pain genetic variants across open chromatin regions of different cell populations from human brain and mouse dorsal horn. For the brain snATAC-Seq data (36), we analyzed the following cell types: astrocytes, hippocampal excitatory neurons, isocortical astrocytes, isocortical excitatory neurons, isocortical inhibitory neurons, microglia, neurons, nigral astrocytes, nigral neurons, nigral OPCs, striatal astrocytes, and striatal inhibitory neurons. For mouse snATAC-Seq data (24), we analyzed the conserved dorsal horn glial and neuronal subtypes. Because of their complexity, multi-allelic variants and those from the MHC region were excluded. Cell types were considered to be significantly associated with chronic pain using an FDR-adjusted *P* value of less than 0.05 (treating human brain snATAC-Seq, mouse spinal snATAC-Seq, or mouse snRNA-Seq datasets separately).

Following previous examples (95, 96), neurotransmitters for the brain snRNA-Seq cell clusters were named on the basis of annotations from Siletti et al. (30). Brain regions were assigned to each cell type according to human brain dissections from the same study. Because cell types are not exclusively derived from a single brain region, we assigned a brain region label to a cell cluster only when most of its cells (>50%, typically much higher) originated from that region. We evaluated the enrichment of significant brain cell types in their neurotransmitters and regions using a hypergeometric test.

### MAGMA enrichment analyses.

We used MAGMA (37) in FUMA (version 1.3.6a) (108) to map chronic pain GWAS SNPs to 19,437 protein-coding genes according to their physical position in NCBI build 37. Genes with nonunique names were removed, as were those in the MHC region (chromosome 6, base positions 25,000,000–34,000,000), where the high linkage disequilibrium (LD) among SNPs creates uncertainty as to which mapped genes account for the observed associations. Cell-type analysis in MAGMA version 1.08 (37, 41) was applied to test for gene enrichment conditioning on the dataset’s average per-gene expression. We used an FDR-adjusted *P* value of less than 0.05 to account for multiple testing and identify significantly enriched cell types (or tissues).

### Gene prioritization for chronic pain across cell types.

We used the LDAK-GBAT package (52) to prioritize genes that contribute substantially to the heritability of chronic pain. To ensure matching of the population LD structure, we used LD reference panels for EUR individuals from 10,000 unrelated UKB participants using LDAK version 5 (52). Exonic and intronic SNPs were mapped to genes on the basis of RefSeq gene annotations (109). We used the default heritability model in LDAK-GBAT to estimate the heritability attributable to individual genes. We determined the significance of heritable genes for chronic pain using an FDR-adjusted *P* value of less than 0.05.

Next, we used fGSEA (53, 110) to identify brain and hDRG cell types with the top-most enriched heritable gene sets. To annotate pathways for chronic pain, we used the gene sets for each enriched cell type as input for ToppGene (54) to test for overrepresentation for GOmolecular functions, GO biological processes, and GO cellular components. Gene sets and pathways with an FDR-corrected *P* value of less than 0.05 were considered significant.

### Statistics.

Statistical analyses were performed in R unless otherwise specified. Data are expressed as associated *P* values and were analyzed using the 1-sample Student’s *t* test and 1-way ANOVA, with the Benjamin-Hochberg FDR to correct for multiple comparisons. A corrected *P* value of less than 0.05 was considered significant. Data in figures represent either average gene expression levels or association *P* values, which were derived from statistical analysis to identify differentially expressed genes. Statistical methods for scRNA-Seq analyses are described separately (see *Calculation of cell-type expression specificity* in Methods). All raw data used to plot figures are presented in Supplemental Tables 1–19.

### Study approval.

Use of human single-cell data for the C1-C2 neck pain study was reviewed and approved by the University of Washington Internal Review Board (IRB no. 10916). Written informed consent was obtained from all patients. Ethics approvals for public datasets are detailed in the original studies.

### Data availability.

All association statistics generated in this study are available within the article and the Supporting Data Values file. The full summary statistics from the GWAS meta-analyses are available upon request to the corresponding author. Each of the summary statistics included in the meta-analyses and the multi-omics datasets included in the enrichment analyses are publicly available.

## Author contributions

HRK and ST conceived the project. ST, MP, MJL, CS, HY, AAT, UFE, CH, MC, WL, ARP, RPS, RLK, TJP, and HRK designed the study and collected data. ST, MP, MJL, and CS performed data analysis. HRK, ARP, RPS, RLK, TJP, and LD supervised the study. ST, MP, MJL, CS, TJP, SGW, and HRK wrote the manuscript. All authors reviewed and approved the final version of the manuscript.

## Funding support

This work is the result of NIH funding and is subject to the NIH Public Access Policy. Through acceptance of this federal funding, the NIH has been given a right to make the work publicly available in PubMed Central.

- US Department of Veterans Affairs grants I50 RX002999-01 (to SGW) and I01 BX003341 (to HRK).
- Veterans Integrated Service Network 4 Mental Illness Research, Education and Clinical Center, Education and Clinical Center.
- NIH grants K99 DA060906 (to ST), F31 NS134318 (to MJL), U19 NS135528 (to WL), R56 NS133364 (to ARP and RPS), K01 AA028292 (to RLK), U19 NS130608 (to TJP, MC, and CPH), and P30 DA046345 (to HRK).

## Supplementary Material

Supplemental data

Supplemental tables 1-19

Supporting data values

## Version 1 10/07/2025

In-Press Preview

## Version 2 12/15/2025

Electronic publication
